# Design, Implementation, and Evaluation of the Adolescents and Surveillance System for the Obesity Prevention Project: Erratum

**DOI:** 10.1097/01.md.0000483088.38677.f8

**Published:** 2016-04-29

In the article “Design, Implementation, and Evaluation of the Adolescents and Surveillance System for the Obesity Prevention Project”,^1^ which appeared in Volume 95, Issue 12 of *Medicine*, Dr. Jemni's institution was incorrectly listed as the University of Greenwich. The correct affiliation is the Department of Sport Science, College of Arts and Sciences, Qatar University, Qatar. The article has since been corrected online.

## References

[1] Tabacchi G Bianco A Alessi N Design, implementation, and evaluation of the adolescents and surveillance system for the obesity prevention project. *Medicine.* 2016 95:e3143. 27015195 10.1097/MD.0000000000003143 PMC4998390

